# Resolution Doubling in 3D-STORM Imaging through Improved Buffers

**DOI:** 10.1371/journal.pone.0069004

**Published:** 2013-07-17

**Authors:** Nicolas Olivier, Debora Keller, Pierre Gönczy, Suliana Manley

**Affiliations:** 1 Laboratory for Experimental Biophysics, School of Basic Sciences, Swiss Federal Institute of Technology (EPFL), Lausanne, Switzerland; 2 Swiss Institute for Experimental Cancer Research (ISREC), School of Life Sciences, Swiss Federal Institute of Technology (EPFL), Lausanne, Switzerland; Julius-Maximilians-University Würzburg, Germany

## Abstract

Super-resolution imaging methods have revolutionized fluorescence microscopy by revealing the nanoscale organization of labeled proteins. In particular, single-molecule methods such as Stochastic Optical Reconstruction Microscopy (STORM) provide resolutions down to a few tens of nanometers by exploiting the cycling of dyes between fluorescent and non-fluorescent states to obtain a sparse population of emitters and precisely localizing them individually. This cycling of dyes is commonly induced by adding different chemicals, which are combined to create a STORM buffer. Despite their importance, the composition of these buffers has scarcely evolved since they were first introduced, fundamentally limiting what can be resolved with STORM. By identifying a new chemical suitable for STORM and optimizing the buffer composition for Alexa-647, we significantly increased the number of photons emitted per cycle by each dye, providing a simple means to enhance the resolution of STORM independently of the optical setup used. Using this buffer to perform 3D-STORM on biological samples, we obtained images with better than 10 nanometer lateral and 30 nanometer axial resolution.

## Introduction

Determining the nanoscale distribution of specific proteins in their cellular context is of paramount importance for understanding their biological function. Super-resolution fluorescence imaging has emerged as an attractive approach to achieve this goal by increasing the resolution of light microscopy by more than a factor of ten [1]. Among super-resolution methods, three-dimensional stochastic optical reconstruction microscopy (3D-STORM) [2] offers among the highest resolutions currently available, by stochastically switching fluorophores between fluorescent and dark states and precisely localizing their positions. Nanoscale lateral (x–y)-localization is generally achieved by determining the centers of single molecule point-spread functions (PSFs), and a number of methods have been developed to precisely determine axial (z) positions. Interferometric detection [3], [4] provides the most precise z-localization achieved to date, but at the cost of intricate custom-built optical setups. Other methods easier to implement include point-spread-function shaping using astigmatism [2] or a double-helix [5], where the axial position of an emitter is determined by the shape of its image on the camera, as well as bi-plane imaging [6] where two parallel planes located at different depths are imaged simultaneously. These simpler methods require a compromise between lateral and axial localization, and typically achieve ∼3-fold lower z- than (x–y)-localization, resulting in anisotropic images where the orientation of the structures of interest can become a limiting factor. A fundamental limit to the localization precision in STORM is the number of photons detected from each fluorophore [7]. Therefore, one way to improve the 3D localization precision is through increased detection efficiency, as was recently demonstrated with a complex optical setup using two opposing lenses to double photon collection [8]. An alternative approach would be to increase the number of photons emitted by each dye. This would allow better localization precision, independent of the microscope setup. In principle, such an increase could be achieved by optimizing the buffers used to control blinking of the dyes [9].

To date, the composition of STORM-buffers has scarcely evolved since the first demonstrations of single dye molecule controlled switching, with a combination of an enzymatic oxygen-scavenging system and a reducing agent (usually a thiol: Mercaptoethilamine –MEA [10], Mercaptoethanol – BME, or recently TCEP [11]) remaining the most widely used [10]–[16]. Here, we show that STORM-buffer optimization using the polyunsaturated hydrocarbon cyclooctatetraene (COT) can provide significantly increased photon yields and therefore localization precision for the dye Alexa-647. In this buffer, a steady blinking is maintained with little irreversible photobleaching, thus allowing high single molecule localization density for super-resolution image reconstruction. This improved buffer allows us to resolve the nanoscale distribution of biological samples on a simple microscope, which we illustrate by performing 2D and 3D STORM of microtubules.

## Results and Discussion

**Figure 1:**
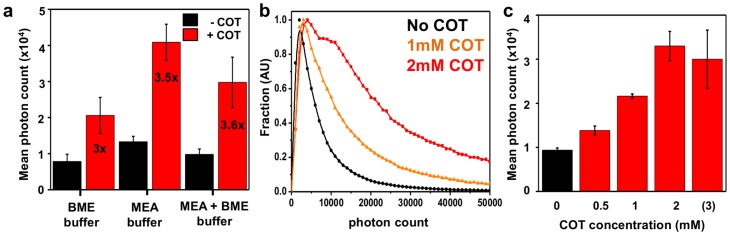
Improving photon counts for STORM with COT. (**a**) Mean photon counts measured for three different STORM buffers using different thiols (MEA and/or BME) and the same oxygen scavenging system (glucose oxidase/catalase, **see Table S1 &** **Methods** for details on composition); number indicates the corresponding fold-increase upon addition of 2 mM COT (red bars) compared to no COT added (black bars) (pH ∼7.5) (**b**) Normalized photon count distributions for the “MEA+BME” buffer as a function of COT concentration (pH 8). (**c**) Mean photon count as a function of COT concentration for the buffer “MEA+BME” (pH 8).

One of the best and most widely used dyes for STORM is Alexa-647 [10], [15], [16]. Because Alexa-647 has been reported in other contexts to be stabilized by cyclooctatetraene (COT) [17], we tested the ability of COT to influence dye brightness under STORM conditions. We performed STORM imaging on immunostained microtubules using different concentrations of COT (**Methods**), and found that COT increased mean molecular photon yields of Alexa-647 up to ∼3.5 fold in a dose-dependent manner, independently of the thiol used (MEA, BME, or both) to induce blinking (**Figure 1a–c**, **Notes S1**). Importantly, this increase came without impairing photoswitching and a high localization density was maintained when adding COT (**Figure S1**). In contrast, Trolox, which was also reported to stabilize Alexa-647 [17], [18], compromised its blinking, thus preventing STORM imaging. Similarly, adding Propyl-Gallate, another commonly used anti-fading agent [19], prevented proper blinking of Alexa-647 (**Table S1**). The fact that COT, but not Trolox, yielded an improvement under STORM conditions sheds light on the probable underlying mechanism. COT has been reported to enhance the stability of dyes through direct quenching of the triplet state by energy transfer [20]. This would explain why the number of photons emitted when the dye is ON is increased without interfering with the redox reaction that puts the dye into a long-lived dark state when the triplet state is reached [9]. By contrast, the stabilizing mechanism of Trolox has been described as a redox reaction [21]. This could interfere with the formation of long-lived dark states by the reducing thiol used in the STORM buffer and thus prevent blinking (**Figure S2**). Since the localization precision scales as the square root of the number of photons detected [7], adding COT to STORM buffers allows an increase in the localization precision by almost a factor 2, independent of the setup used.

**Figure 2:**
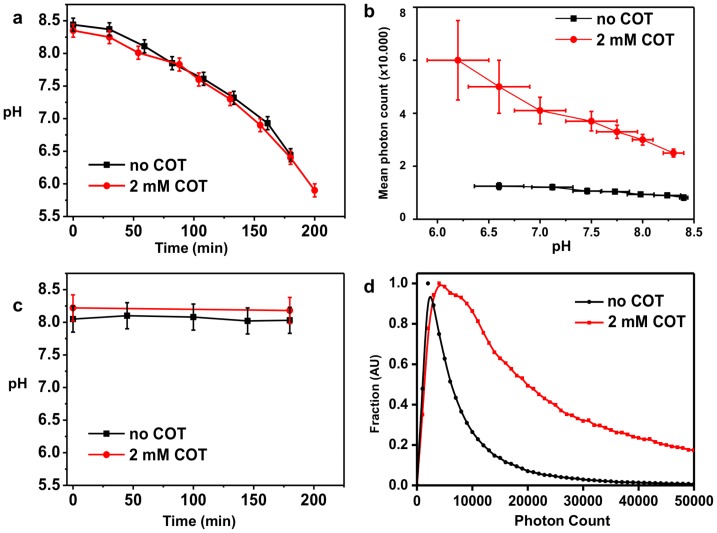
Influence of pH on STORM imaging. (**a**) Decrease in pH as a function of time for the “BME+MEA” buffer containing glucose oxidase/catalase as oxygen scavenging system, (buffer #3 in **Table S1**) both with and without the addition of 2 mM COT. (**b**) Average photon counts per molecule as a function of pH for the “BME+MEA” buffer both with and without the addition of 2 mM COT. (**c**) pH as a function of time using the PCA/PCD oxygen scavenging system both with and without the addition of 2 mM COT (**d**) Normalized photon count distribution for the PCA/PCD buffer with and without COT at pH = 8 (mean photon count is 8,700 without COT and 32,000 with 2 mM COT).

We noticed however a significant variability in the photon counts when performing measurements, and investigated its origin (**Notes S1**). An important component of STORM buffers is an enzymatic oxygen scavenging system, and the most commonly used one – also used for our experiments reported in **Figure 1**– consists of a combination of glucose, glucose oxidase and catalase enzymes. Since this enzymatic system results in a time-dependent acidification of the buffer [22] (**Figure** **2a**), we investigated the pH dependence of Alexa-647 photon counts both with and without COT. As shown in **Figure 2b**, the mean photon count increased significantly in both cases as the pH dropped, but this increase was accompanied by detrimental changes in the blinking properties (**Figure S3**), which prevented the localization of sufficient densities of molecules to form a good STORM image. Therefore, we sought to control pH stability to allow more meaningful comparisons between buffers. We show, for example, that the recently reported enzyme pyranose oxidase [23] can be used to replace glucose oxidase, resulting in a pH-stable buffer (**Figure S4**). Another alternative to the above-mentioned oxygen scavenging systems is the combination of protocatechuic acid and protocatechuic dioxygenase (PCA/PCD) [24], which enabled imaging over several hours without any detectable changes in pH (**Figure** **2c**). We ensured that the single molecule fluorescence properties of Alexa-647 in the buffer with PCA/PCD are equivalent to those measured in glucose-oxydase/catalase at the same pH, and that the addition of COT to this buffer also yielded a ∼3.5-fold increase in brightness (**Figure 2d**). We subsequently used this oxygen scavenging system for STORM imaging of microtubules.

**Figure 3:**
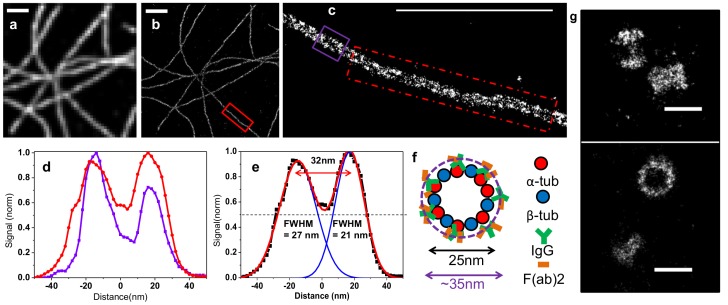
Buffer-Enhanced 2D STORM imaging of microtubules. (**a**) Widefield, and (**b**) STORM image of a COS-7 cell stained with alpha-tubulin primary and Alexa-647-F(ab’)2 secondary antibodies, imaged in Buffer #4 (see **Table S1**). (**c**) Zoom on the ROI (red box) defined in (b), number of localized molecules = 1960. (**d**) Lateral profiles taken either from a 200 nm-wide region (violet box) and corresponding curve shown in violet, or averaged over seven 200 nm-wide regions (inside the dashed red box) highlighted in (c) and the corresponding curve in red. (**e**) Averaged lateral profile shown in (d) fitted with a double Gaussian. (**f**) Model of the stained microtubule (see also **Figure S4**). The labeled antibodies are expected to form a ring around the microtubule with an inner diameter of ∼25 nm and an outer diameter of ∼50 nm. (**g**) STORM image of COS7 cells strained with Cep152 primary and Alexa-647 F(ab’)2 secondary antibodies, imaged in Buffer #4 (see **Table S1**). The top panel shows two tori from a side view, the bottom panel one torus in side views and one torus in cross-section. Scale bar is 1000 nm for (**a–c**) and 500 nm for (**g**).

Microtubules are hollow cylindrical structures with a diameter of 25 nm [25], whose minute size makes them popular test samples for super-resolution microscopy. We immunostained alpha-tubulin, one of the two proteins constituting microtubules, using primary antibodies that decorate the outer part of the cylinder, and secondary antibody fragments labeled with Alexa-647. While previous STORM imaging using full length secondary antibodies resolved the hollow inner spaces of microtubules in only a few regions [16], we show here that the increased resolution afforded by the enhanced buffer allowed us to do so over the entire image (**Figure 3** & **Figure S5**). We measured a distance of ∼32 nm, between tube walls on both small (200 nm, blue) and large regions (1400 nm, red). This size is consistent with a microtubule diameter of 25 nm augmented on each side by ∼10 nm of antibodies (**Figure 3f** & **Figure S6**). The high photon counts combined with steady photoswitching rates allowed us to use only the brightest and therefore best-localized molecules to reconstruct our images; for instance, when we filtered out all molecules localized with less than 5,000 photons we still acquired a density of ∼1.4 molecules per nm (**Figure 3b** & **Methods**). We then set out to ensure that this new buffer was versatile enough to image cellular structures of different sizes and protein densities. The pericentriolar protein Cep152 appeared as a good candidate to this end, because its organization within the proteinaceous matrix surrounding the centrioles has recently been reported using 3D-SIM microscopy to be a torus ∼435 nm in diameter [26]. We performed 2D STORM on Cep152 using our optimized buffer (**Figure 3g**) and could likewise visualize a torus-like arrangement at centrosomes, consistent with the published super-resolution images [26].

**Figure 4:**
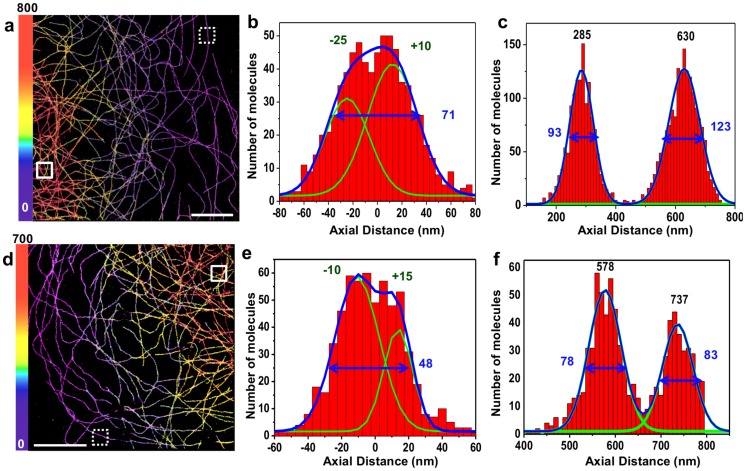
Buffer-Enhanced 3D STORM imaging of Microtubules. COS-7 cells were stained with alpha-tubulin primary antibodies and Alexa-647 F(ab’)2 secondary antibodies and imaged. 3D-STORM images are color coded by depth.(**a–c**) Astigmatic 3D-STORM with an oil objective and PBS-Glycerol buffer (Buffer #5, see **Table S1**)**:** (**a**) 3D-STORM image and corresponding axial profiles from 300×300 nm-wide regions taken (**b**) on the edge of the cell (dashed box) or (**c**) in a denser central region with microtubules crossings (full box).(**d-f**) Astigmatic 3D-STORM with a water objective and an index-matched buffer (Buffer #4, see **Table S1**)**:** (**d**) 3D-STORM image and corresponding axial profiles from the 300×300 nm-wide regions taken (**e**) on the edge of the cell (dashed box) or (**f**) in a denser central region with microtubules crossings (full box). For each axial profile, positions of the fitted Gaussian peak maxima (green) as well as FWHM (blue) are indicated. Scale bar is 5 µm.

We next tested the COT-enriched buffer for 3D-STORM imaging on a custom-built single-objective microscope to which we introduced astigmatic aberrations with a cylindrical lens for axial localization. We first imaged microtubules using an oil objective, usually used in STORM because of their high numerical aperture (NA), and added glycerol to our buffer to minimize index mismatch [27] (**Figure S7** & **Methods**). We used two measures for our axial localization precision: vertical profiles of microtubules, to see whether their hollow structure could also be detected here, as well as repeated localizations of single molecules (**Figure S8**). We estimated our mean axial localization precision from repeated localizations to be ∼32 nm (FWHM) axially, insufficient to resolve the hollowness of the microtubules though still enough to observe a deviation from a Gaussian profile along the z-axis (**Figure 4b**). However, the aberrations due to the index mismatch still limited our resolution significantly, especially at greater depths (**Figure 4c**).

To reduce the effect of aberrations, we switched to imaging with a water immersion objective, and did not add any glycerol. The smaller NA (1.2) of this objective means lower localization precision for a given photon count [7], but it has the advantage of being almost perfectly index-matched with the buffer. This tradeoff is expected to yield a positive gain due to the marked increase in photon yields afforded by our buffer. We once again assessed the performance of the system with microtubule images. As shown in **Figure 4e** **,** not only can the hollowness of microtubules be inferred along the z-axis at the edge of the cell, but microtubules far from the coverslip have the same apparent size as those closer to the coverslip (**Figure 4f**); thus, the resolution is not affected by depth within this range (>500 nm). From these images, we deconvolved the known size of the structure (determined by EM and by 2D imaging in **Figure 3**) from our axial and lateral profiles, and estimated our localization precision to be ∼5–10 nm laterally and 25–30 nm axially in FWHM (**Figure S5**). We also used repeated single molecule localizations from our images, and obtained a representative distribution of the localization precision (**Figure S8**), with average values of ∼9 nm laterally, and 25 nm axially (FWHM) with this water objective. We stress out that removing more of the low photon-count peaks would reduce these values, but at the same time would lower the peak density in the final image.

**Figure 5:**
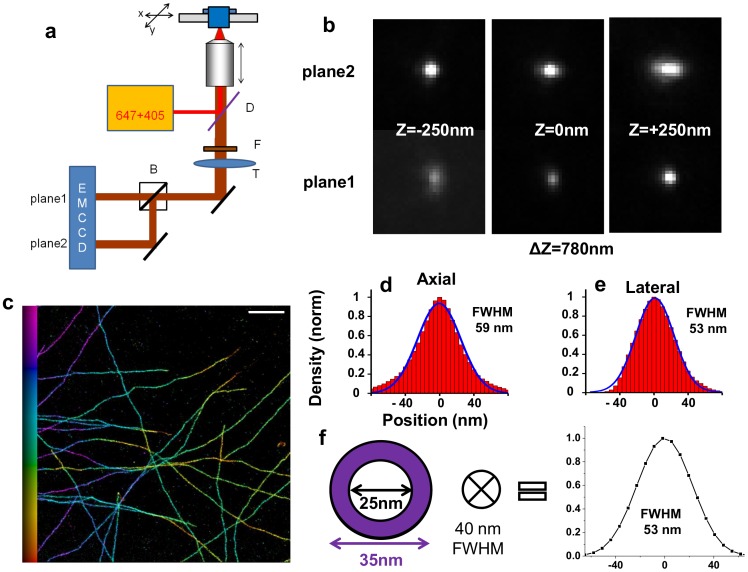
Buffer-Enhanced 3D STORM imaging on a biplane astigmatic microscope. (**a**) Schematics of the SR-200 inverted microscope (Vutara, Salt Lake City, UT). D: Dichroic mirror, F: Fluorescence filter, T: Tube lens, B: 50/50 Beamsplitter. Fluorescent light from a single molecule is collected by the objective and is imaged onto two different planes located on distinct parts of the EMCCD camera. The distance between the two planes is set by the optical path difference, and results in a measured axial shift of 780 nm. Optical aberrations are represented by in the schematics by the lateral shift in the position of the tube lens. (**b**) Measured PSF in the two planes which are used for 3D localization, shown at three different depths. (**c**) Biplane-Astigmatic 3D STORM image of a cell stained with Alexa-647-conjugated alpha-tubulin antibodies, color-coded by depth. Buffer #4 is used (See **Table S1**) (**d,e**) Lateral and axial profiles were measured and averaged from five 200-nm wide regions. The blue line corresponds to a Gaussian fit, and the corresponding FWHM value is indicated. (**f**) Estimation of the resolution performed by convolving the projection of the known structure with a 40 nm wide Gaussian function, and the resulting expected distribution. Scale bar is 1 µm for (**b**) and 2.5 µm for (**c**).

Finally, we tested whether we could improve the isotropy of our image, since even with the improved photon count afforded by our buffer, the axial localization precision remains significantly lower that the lateral one. We used a 3D commercial microscope based on biplane imaging [6], and we introduced additional astigmatic aberrations to increase the z-dependence of the PSF and therefore reduce the anisotropy of the localization precision (**Figure 5a–b**). This time, we imaged microtubules stained with antibodies directly labeled with Alexa-647, which results in a slightly thinner labeled structure (**Figure 5c**). The resulting profiles measured at the edge of the cell yielded lateral and axial sizes within a few nanometers of one another (53 nm and 59 nm respectively, **Figure 5d–e**) showing that using this buffer on commercial systems enables an almost isotropic 3D resolution of ∼40–50 nm (**Figure 5f**). While the absolute resolution is slightly decreased compared to that obtained on the home built hardware – which can be attributed to differences in the system (excitation wavelength, filters, imperfect control of the aberrations) – the almost isotropy of this system makes it promising for many applications.

### Conclusions

Overall, we have used the enhancement of single fluorophore brightness to significantly improve STORM resolution, as demonstrated by our determination of the nanoscale distribution of alpha-tubulin in microtubules. Although superior axial and radial resolutions have been previously reported for 3D super-resolution imaging [3], [4], [8], the resolution achieved on the fairly simple microscope setups used here should be widely accessible, and thus enable the broader scientific community to address other biological questions at the nanoscale. Importantly, the use of COT-enhanced buffers has the distinct advantage of being generally transferrable, and to improve the achievable resolution as well as to relax the technical requirements for STORM. This brings the performance of commercial microscopes to the level of custom-built systems and also extends the range of possibilities achievable with complex setups. While we only show single color imaging here, we expect that multicolor imaging using activator-reporter pairs [28] with Alexa-647 used as the reporter is possible. Moreover, since COT has been shown to increase the stability of several cyanide dyes [17], it has the potential of also increasing the brightness of these dyes under their adapted STORM conditions.

Our results emphasize that improving the imaging buffers can be a general strategy for increasing resolution, to be considered in parallel to instrument development, as has recently been demonstrated for several super-resolution techniques [10], [29], [30]. Moreover, recent studies have shown that COT could be directly conjugated to dyes to increase their stability [31], [32], and these dyes have the potential of providing even higher resolution.

## Materials and Methods

### Sample Preparation and Immunofluorescence Staining

African green monkey kidney cells (COS-7) were cultured in DMEM supplemented with 10% FBS (Sigma-Aldrich) in a cell culture incubator (37°C and 5% CO2). Cells were plated at low confluency on cleaned 25 mm #1.5 coverglass coated with gold fiducial markers (Hestzig –600-30 AuF) for imaging, and 25 mm #1 coverglass (Menzel) for photon-counting experiments. Prior to fixation, all solutions were pre-warmed to 37°C: 24 h after plating, cells were pre-extracted for 30 s in 0.5% Triton X-100 (Triton) in BRB80 (80 mM PIPES, 1 mM MgCl_2_, 1 mM EGTA, adjusted to pH 6.8 with KOH) supplemented with 4 mM EGTA, washed in PBS, fixed for 10 min in −20°C Methanol (Sigma-Aldrich), then washed again in PBS. The samples were then blocked for 30 minutes in 5% BSA, before being incubated for 1.5 h at room temperature with 1∶1000 mouse alpha-tubulin antibodies (Sigma, T5168) in 1% BSA diluted in PBS −0.2% Triton (PBST), followed by 3 washes with PBST, and then incubated for 45 min in 1%BSA-PBST with 1∶1000 goat anti-mouse Alexa Fluor 647 (Alexa-647) F(ab’)2 secondary antibody fragments (Life Technologies, A-21237). For **Figure 5** **,** the same protocol was used except that cells were plated on 18 mm #1.5 coverslips (LH23.1, Carl Roth), and that instead of primary and secondary antibodies, mouse anti-alpha-tubulin antibodies were directly conjugated using the APEX Alexa Fluor 647 antibody labeling kit (Life Technologies) according to the manufacturer’s instructions. For Cep152 imaging, U2OS cells (European Collection for Cell Cultures) were maintained in McCoy’s 5A GlutaMAX medium (Life Technologies) supplemented with 10% FBS in a cell culture incubator (37°C and 5% CO2) and plated at low confluency on cleaned 25 mm #1 cover-glass (Menzell). Fixation and immunostaining was performed similarly as for tubulin, except that the primary rabbit anti-Cep152 antibody (Sigma-Aldrich, HPA039408) was used at 1∶2000 in 1% BSA - PBST, and the secondary antibody was goat anti-rabbit Alexa-647 F(ab’)2 secondary antibody fragments (Life Technologies, A-21246) at 1∶1000 in 1% BSA - PBST.

### Imaging Buffers Preparation

Mercaptoethylamine (MEA – Sigma-Aldrich 30070) was prepared as a 1 M stock solution in deionized water, then adjusted to ∼pH 8 using glacial acetic acid (Sigma-Aldrich), and stored at 4°C. β-mercaptoethanol (BME - Sigma-Aldrich M6250) was stored undiluted (14.3 M) at 4°C. Cyclooctatetraene (COT – Sigma-Aldrich 138924) and Trolox (Sigma-Aldrich 238813) were reconstituted in pure DMSO as 200 mM stock solutions. PCA (Protocatechuic acid, Sigma-Aldrich 37580) was dissolved to 100 mM in deionized water and adjusted to pH 9 using KOH and stored at 4°C; PCD (Protocatechuic dioxygenase, Sigma-Aldrich P8279) was stored at −20°C in 50% glycerol in 50 mM KCl, 1 mM EDTA and 100 mM Tris-HCl pH 8 at a concentration of 5 µM [24]. Propyl Gallate (Sigma-Aldrich P3130) was prepared as a stock solution of 16 mM in PBS. Pyranose Oxidase (Sigma-Aldrich P4234) was kept at −20°C, and weighed before being added to the buffer.

Imaging and associated photon-counting was performed in a solution containing 10% (w/v) glucose in 10 mM PBS-Tris pH 7.5 with 10 mM MEA combined with 50 mM BME, 2 mM COT, 2.5 mM PCA and 50 nM PCD. Glucose was removed for 3D imaging with the 60× objective (see **Optical setup**).

Photon-counting experiments were also performed using three other STORM imaging buffers: 10 mM Tris (pH 7.5), oxygen scavengers (0.2 mg/mL glucose oxidase (Sigma-Aldrich G0543-10KU), 57 µg/mL catalase (Sigma-Aldrich C3515) and either 100 mM MEA (‘MEA buffer’), 100 mM BME (‘BME buffer’), or 10 mM MEA combined with 50 mM BME (‘MEA+BME buffer’). Oxygen scavengers, thiols and COT solutions were diluted to the indicated concentrations approximately one hour prior to imaging, and the buffer was then adjusted to pH ∼8 with 1 M HEPES (Sigma-Aldrich H3662) before imaging (typical HEPES concentration in the buffer: ∼25 mM).

Imaging for **Figure 4a–c** was performed in a 25% 10 mM TRIS-PBS-75% Glycerol solution containing 10 mM MEA, 50 mM BME, 2 mM COT, 2.5 mM PCA and 50 nM PCD, with a measured index of refraction of 1.44 (see below).

Imaging for **Figure S3** was performed in a 10 mM Tris-PBS pH 8+10% Glucose solution containing 10 mM MEA, 50 mM BME, 2 mM COT, 57 µg/mL catalase, and 5 U/mL Pyranose Oxidase.

All the different buffers used are summarized in **Table S1.**

### pH Measurements

pH measurements with the glucose-oxydase/catalse oxygen scavenging system (**Figure 2**, **Figure S3**) were done using 2 mL of “MEA+BME” buffer. The buffer was prepared as previously described, the pH was then measured prior to imaging (Microprocessor pH meter, Hanna Instruments). Three STORM images were then taken (each 10,000 frames at 25 frames per second (fps), corresponding to 20 minutes), and the mean photon count from each measurement was extracted. The error bars plotted correspond to twice the standard deviation between the 3 measurements. After the measurements, the pH of the buffer was re-measured, and the value of the pH before and after imaging was averaged. Imaging was then repeated until the pH dropped below ∼6.5. For PCA-PCD (**Figure 2c**), the pH measurement was performed before and after 3 consecutive hours of imaging. For Pyranose Oxidase (**Figure S4**), pH was measured both before and after 2 hours of imaging, and no difference in pH could be detected.

### Optical Indices Measurement

Optical indices measurements were performed at 594 nm with a digital refractometer (KRUSS, DR210-95).

### Optical Setup

Imaging as well as single molecule photon counting was performed on a modified Olympus IX71 inverted microscope. A 641 nm laser (Coherent, CUBE 640-100C) and a 405 nm laser (Coherent, CUBE 405-100C) was reflected by a multiband dichroic (89100 bs, Chroma) on the back aperture of a 100×1.3 NA oil objective (Olympus, UplanFL) or 60×1.2 NA water immersion objective (Olympus UPLSAPO 60XW) (for **Figure 4d–f** and **Figure S7d–f**) mounted on a piezo objective scanner (P-725 PIFOC, Physik Instrumente). The collected fluorescence was filtered using a band-pass emission filter (ET700/75, Chroma) and imaged onto an EMCCD camera (IxonEM+, Andor) with a 100 nm pixel size (167 nm for the 60×Objective) and using the conventional CCD amplifier at a frame rate of 25 fps. Laser intensity on the sample measured after the objective was ∼2–4 kW.cm^−2^ with the 100× objective, and ∼1 kW.cm^−2^ with the 60× objective. 10,000–20,000 frames were recorded for the photon-counting experiments, and 15,000–20,000 for the imaging experiments.

3D imaging (**Figure 4**) was performed by adding a cylindrical lens to the imaging path, using one arm of an Optosplit system (CAIRN). The cylindrical lens (f = 1000 mm, Throlabs LJ1516RM-A) was added at the position of the fluorescence filter, which is close to the Fourier plane.

3D STORM imaging (**Figure 5**) was performed on a SR-200 inverted microscope (Vutara, Salt Lake City, UT) based on the biplane approach [6] using a 60×/1.42 NA oil objective (Olympus, UIS2 PLANAPO). Extra magnification was used to achieve a pixel size of 101 nm on an EMCCD camera (Photometrics). A 647 nm laser (Coherent) was used for excitation, with a power of ∼4.5 kW.cm^−2^, and a 405 laser (Coherent, CUBE) for re-activation (few mW.cm^−2^). Data was recorded at 25 fps, and the acquisitions consisted of 20,000 raw images. Raw data was analyzed by the Vutara SRX software (v4.04). In brief, particles were identified by their brightness from the combined images taken in both planes simultaneously. If a particle was identified in multiple subsequent camera frames, data from these frames was summed up for the specific identified particle. Particles were then localized in three dimensions by fitting the raw data in a 16×16 pixel region of interest centered on each particle in each plane with a 3D reference obtained from recorded bead calibration data sets. Sample drift was corrected by cross-correlation of the localized particles according to [33].

### Data Analysis

Photon counting experiments under STORM conditions (**Figure 1a** **, Figure S1 & S3**) were performed on immunostained tubulin samples (see **Sample preparation and immunofluorescence staining**). Each peak with a high enough signal-to-noise ratio was fitted to a Gaussian function and analyzed, and photon counts were extracted from the fitted peaks (Peakselector, courtesy of H. Hess) using the camera sensitivity (***s***) determined from a mean signal vs. variance plot [34]. Outliers (peaks detected for more than 15 consecutive frames, and peaks not fitted properly with a Gaussian function) as well as peaks localized with less than 1000 photons were removed from the analysis. Histograms of photons per molecule were normalized by dividing all bins by the number of molecules with the mode value. Peaks detected in successive frames at a distance of less than 40 nm were grouped and considered as a single molecule (see paragraph below for grouping details).

Grouping of successive localizations was performed using Matlab. Localized peaks were tracked in 2D (x–y) using a single particle tracking algorithm (http://physics.georgetown.edu/matlab/index.html ) with a maximum search radius of 40–50 nm, and all the localizations in a track were averaged to give a final molecular location, as well as molecular number of photons. The standard deviation of the *x*, *y*, and *z* position were also calculated for each track, and used for **Figure S7**. Molecules displaying unusually large standard deviation (especially in z) were discarded from the analysis.

For 2D STORM (**Figure 3**), peaks were localized using Peakselector, and those detected in successive frames at a distance of less than 40 nm were grouped and considered as a single molecule. De-drifting was performed by subtracting from the localized peak positions the displacement of a fiducial marker whose position was determined using a rolling average of 200 frames. All the molecules with fewer than 5000 detected photons were discarded. The data was rendered using Matlab (‘hist3’ function) by binning the localizations in a 2 nm pixels grid, and then a Gaussian blur of 2 nm sigma was added to obtain a smoother rendering using ImageJ.

For astigmatic 3D STORM (**Figure 4**), the width and height (x- and y- dimensions) of the image of a single emitter as a function of depth was calibrated using fluorescent beads, according to [2], [27]. Briefly, images of ∼10 beads were recorded at successive depths by using the objective piezo scanner with steps of 20 nm, and fitted with an elliptical Gaussian function using Peakselector. The width vs. depth and height vs. depth of each bead was then fitted with a model function [2] using Matlab’s “fit” function and the fit was averaged between the different beads to give a calibration curve. A look-up-table (**Figure S8a,d**) was then created from this calibration data for every combination of width and height by minimizing the distance between the measured height and width and the calibration data with Matlab’s “fminsearch” function, with the resulting minimum value used as a goodness of fit and saved in another look-up-table (**Figure S8b,e**). Finally, this look-up table was used to convert the measured width and height parameters from the fitted peaks into a *z*-coordinate. Peaks localized with a goodness of fit lower than an arbitrary threshold were discarded from the analysis. The data was then grouped using Matlab, and the final localizations were rendered using Peakselector, with the depth color-coded.

## Supporting Information

Figure S1 **Blinking in the presence of COT.** Analyses performed on an immunostained tubulin sample showing **(a)** Number of detected molecules in a 128×128 pixel region (pixel size 100 nm) over 64.000 frames with only the 641 nm laser ON (∼4 kW/cm^2^) using buffer #4 (see **Table S1**); **(b)** Number of detected molecules in a 128×128 pixel region over 64.000 frames with both 641 nm and 405 nm lasers ON (∼4 kW/cm^2^ and ∼1 W/cm^2^) using buffer # 4; **(c)** Number of detected molecules in a 64×64 pixels region over 40.000 frames with 641 nm laser ON, and adding the 405 nm laser after 14.000 frames. (∼4 kW/cm^2^) using buffer #4.
(TIFF)

Figure S2 **Mechanism of Alexa-647 blinking.** Energy diagram of Alexa-647 under the influence of the different chemicals used in this article. STORM blinking relies on the cycling between the ON state, and the reduced OFF state (long-lived dark states), and is achieved by adding a reducing agent in an oxygen-depleted environment [9]. Adding COT to this buffer improves the lifetime of the ON time through direct energy exchange with the triplet state [20], while adding Trolox acts on both the ON state through its reduced form (TX), and on the dark state trough its oxidized form (TQ) [21], thereby interfering with the proper cycling of the dye. Adapted from [15].
(TIFF)

Figure S3 **Influence of the pH on the mean number of photons emitted and experimental considerations. (a)** Mean photon count vs. pH for STORM buffer #3 (“BME+MEA”, see **Table S1)** with (red curve) and without (black curve) COT; three different regions are highlighted: blue (pH >8), green (8>pH >6.75), and red (pH <6.75) - corresponding to different average ON times (τ_on_), which were determined by calculating the average number of frames a given single molecule was on. **(b)** Representative single molecule traces over 5 frames (1 frame = 40 ms, laser intensity ∼4 kW/cm^2^). As the pH decreases, the average ON time of Alexa-647 increases, and while this is at first advantageous because of the increased brightness (compare brightness of peaks in the green box versus the blue box), it becomes problematic at pH lower than ∼6.5 since the ON time becomes very large, leading to a higher probability of overlapping peaks. At low pH values, one way to decrease τ_on_ would be to increase the laser power, but laser powers are limited: on the commercial microscope used for 3D imaging, the maximum intensity at the focus is equal to 4.5 kW.cm^−2^, thus imaging was typically performed in the pH range from ∼7–8. The OFF time were also probably affected, but STORM images only provide us with a direct measurement of the ON times. This behaviour is consistent with single molecule measurements previously reported [35].
(TIFF)

Figure S4 **Imaging properties using Pyranose Oxidase. (a)** 2D STORM image of a COS-7 cell stained with alpha-tubulin primary and Alexa-647 F(ab’)2 secondary antibodies in “Pyranose Oxidase [23] Buffer” (Buffer #6 in **Table S1**), and **(b)** Zoom on the boxed region defined in (a) showing the hollowness of microtubules. **(c)** Lateral profile measured and averaged over a 500 nm-long microtubule region (green box in (b)) and indicated FWHM values as well as distance between two peaks, showing distances consistent with **Figure 3e**. **(d)** Normalized frame (red) and grouped molecules (black) photon count distribution, as well as average values given in the top right corner. **(e)** Normalized frame localization precision in the lateral direction estimated from the dataset shown in **(a)** by calculating the standard deviation of repeated localizations (threshold of 5 localizations). The actual localization precision is expected to be closer to the standard error of the mean, but using only peaks on for more than 5 frames would skew the value.
(TIFF)

Figure S5 **High resolution STORM image of microtubules.** STORM image of the data used for **Figure 3a–f** is displayed by binning the grouped localizations into a regular grid of 3×3 nm. A value of *n* corresponds to *n* molecules per pixel.
(PNG)

Figure S6 **Model of microtubules nanostructure. (a)** Schematics of the cross-section of an immuno-labeled microtubule. The epitope of the alpha-tubulin antibody used is located towards the C-terminal end of the protein (manufacturer's datasheet), which was shown by various electron-microscopy studies to be located outside of the tube (reviewed in [25]). We then assume that the primary antibodies are decorated isotropically with smaller secondary antibody fragments, resulting in a hollow labeled tubular structure with respective inner and outer diameter of ∼25 nm, and ∼50 nm. **(b)** Expected signal from the projection of this 25–50 nm tube assuming different “resolution” (Gaussian blurring of 1–15 nm indicated in sigma). **(c)** Lateral profile from **Figure 3d** (black line) compared with the modeled structure assuming a blurring factor of 4 nm (red line) **(d)** axial profile from **Figure 4e** (black line) compared with the modeled structure assuming a blurring factor of 9 nm (red line).
(TIFF)

Figure S7 **Photon counts & localization precision. (a)** Normalized frame and molecule photon counts distribution extracted from the dataset shown in **Figure 4a–c** **(**Buffer #4 in **Table S1)**, mean values are indicated in the top right corner. **(b)** Frame localization precision in both axial and lateral directions estimated from the dataset shown in **Figure 4a–c** by calculating the standard deviation of repeated localizations (threshold of 5 localizations, see **Notes S1** for more details). **(c)** Frame and molecule photon counts distribution extracted from the dataset shown in **Figure 4d–f** **(**Buffer #5 in **Table S1**), mean values are indicated in the top right corner. **(d)** Frame localization precision in both axial and lateral directions estimated from the dataset shown in **Figure 4d–f** by calculating the standard deviation of repeated localizations (threshold of 5 localizations).
(TIFF)

Figure S8 **Astigmatism z-calibrations. (a)** Estimated z-localization in nm for each couple of (width: wx, height: wy) expressed in pixels between a minimum and a maximum value for the 100× objective obtained from the calibration data (see **Data Analysis** for more details). **(b)** Estimated error in the localization associated to the matrix shown in (a). **(c)** Measured z position vs. measured width and height for the dataset shown in **Figure 4a** (corresponding region in (a) illustrated by the dotted square). **(d)** Estimated z localization in nm for each couple of (width: wx, height: wy) expressed in pixels between a minimum and a maximum value for the 60x water objective obtained from the calibration data. **(e)** Estimated error in the localization associated to the matrix shown in (d). **(f)** Measured z position vs. measured width and height for the dataset shown in **Figure 4e** (corresponding region in **(**d**)** illustrated by the dotted square).
(TIFF)

Table S1 **Buffers used in this study.**
(DOCX)

Data S1 **List of the localized peaks after grouping used to build figure S5.** The first column correspond to the molecule index, the second one correspond to the last frame in which a molecule appears, and the 3^rd^ and 4^th^ column correspond to the x and y localization, in nanometer.
(MAT)

Notes S1 **Discussion on the factors affecting the variability of STORM measurements.**
(DOCX)
