# Supplementary figures and images for: Resolution Doubling in 3D-STORM Imaging through Improved Buffers
Source: PLoS One. 2013 Jul 17;8(7):e69004. doi: 10.1371/journal.pone.0069004 (PMC3714239; doi:10.1371/journal.pone.0069004)

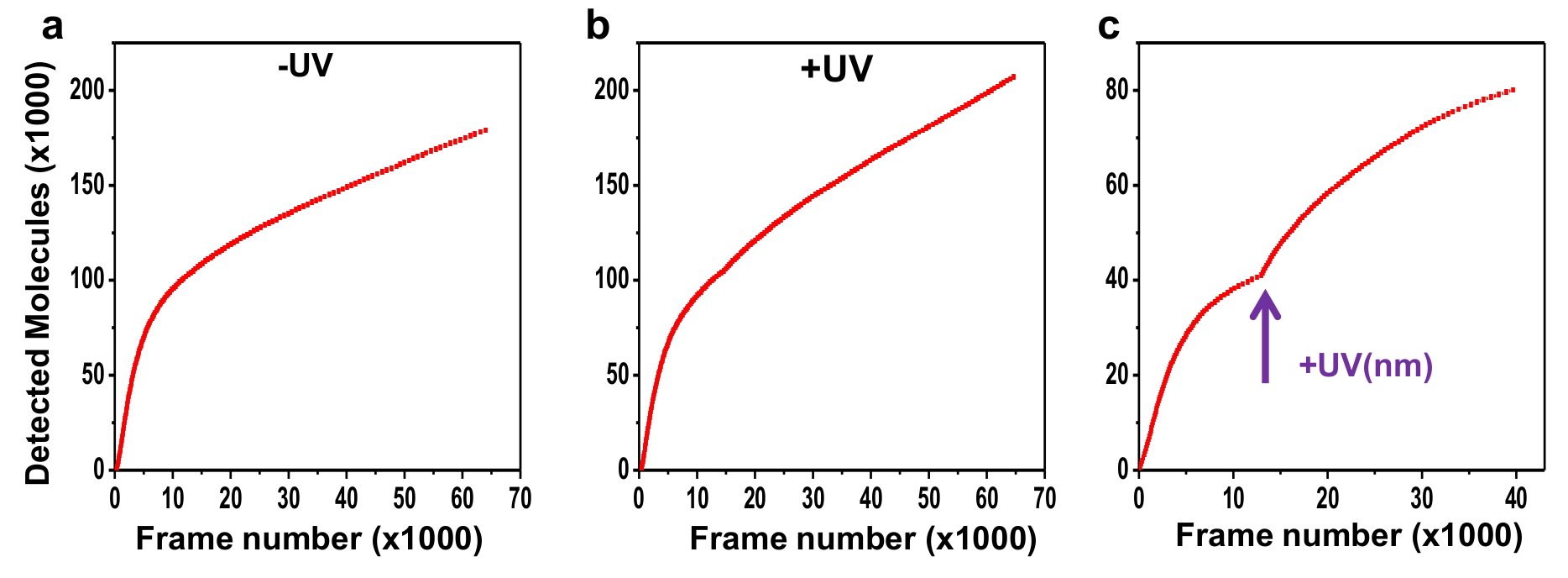

Supplement: Figure S1 — Blinking in the presence of COT. Analyses performed on an immunostained tubulin sample showing (a) Number of detected molecules in a 128×128 pixel region (pixel size 100 nm) over 64.000 frames with only the 641 nm laser ON (∼4 kW/cm2) using buffer #4 (see Table S1); (b) Number of detected molecules in a 128×128 pixel region over 64.000 frames with both 641 nm and 405 nm lasers ON (∼4 kW/cm2 and ∼1 W/cm2) using buffer # 4; (c) Number of detected molecules in a 64×64 pixels region over 40.000 frames with 641 nm laser ON, and adding the 405 nm laser after 14.000 frames. (∼4 kW/cm2) using buffer #4. (TIFF) [file pone.0069004.s001.tiff]

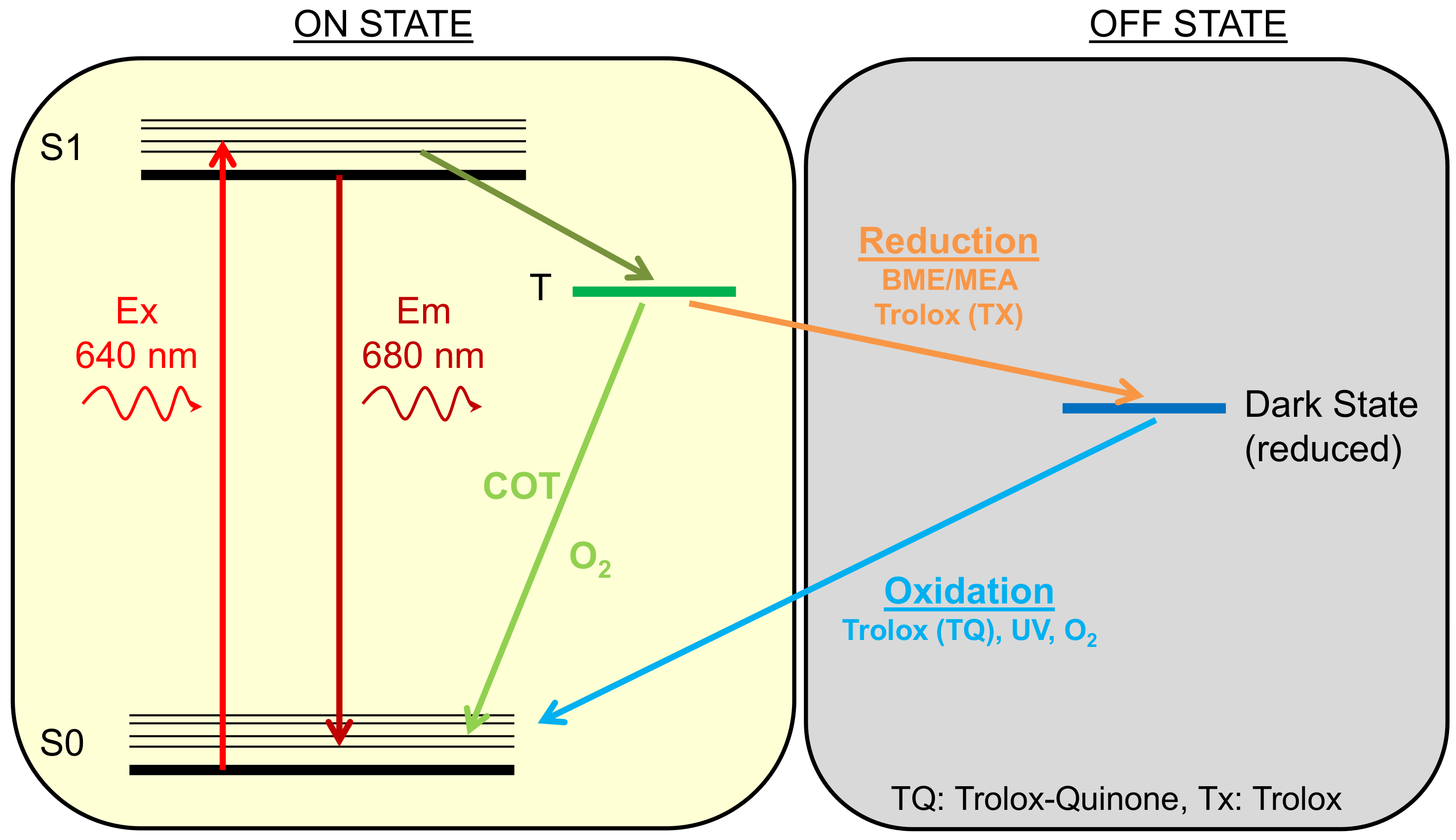

Supplement: Figure S2 — Mechanism of Alexa-647 blinking. Energy diagram of Alexa-647 under the influence of the different chemicals used in this article. STORM blinking relies on the cycling between the ON state, and the reduced OFF state (long-lived dark states), and is achieved by adding a reducing agent in an oxygen-depleted environment [9]. Adding COT to this buffer improves the lifetime of the ON time through direct energy exchange with the triplet state [20], while adding Trolox acts on both the ON state through its reduced form (TX), and on the dark state trough its oxidized form (TQ) [21], thereby interfering with the proper cycling of the dye. Adapted from [15]. (TIFF) [file pone.0069004.s002.tiff]

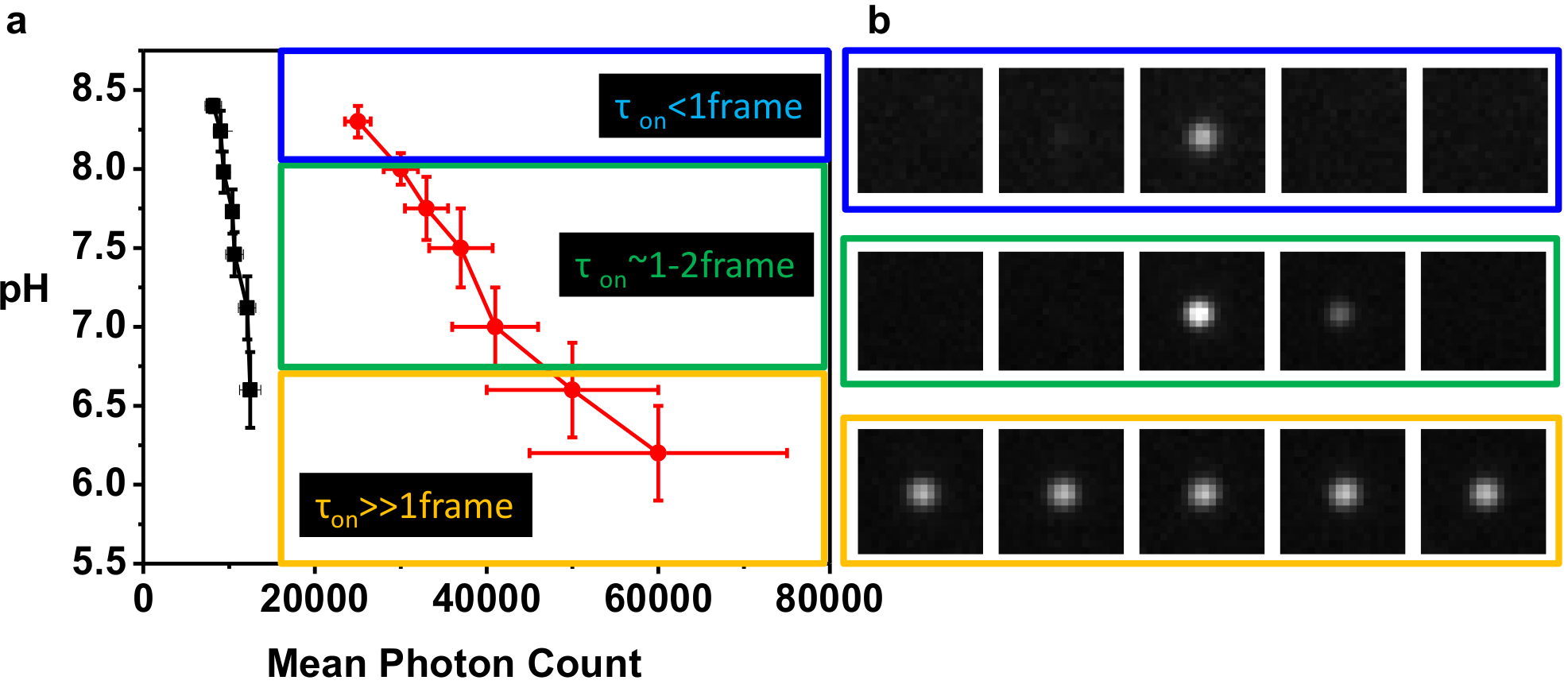

Supplement: Figure S3 — Influence of the pH on the mean number of photons emitted and experimental considerations. (a) Mean photon count vs. pH for STORM buffer #3 (“BME+MEA”, see Table S1) with (red curve) and without (black curve) COT; three different regions are highlighted: blue (pH >8), green (8>pH >6.75), and red (pH <6.75) - corresponding to different average ON times (τon), which were determined by calculating the average number of frames a given single molecule was on. (b) Representative single molecule traces over 5 frames (1 frame = 40 ms, laser intensity ∼4 kW/cm2). As the pH decreases, the average ON time of Alexa-647 increases, and while this is at first advantageous because of the increased brightness (compare brightness of peaks in the green box versus the blue box), it becomes problematic at pH lower than ∼6.5 since the ON time becomes very large, leading to a higher probability of overlapping peaks. At low pH values, one way to decrease τon would be to increase the laser power, but laser powers are limited: on the commercial microscope used for 3D imaging, the maximum intensity at the focus is equal to 4.5 kW.cm−2, thus imaging was typically performed in the pH range from ∼7–8. The OFF time were also probably affected, but STORM images only provide us with a direct measurement of the ON times. This behaviour is consistent with single molecule measurements previously reported [35]. (TIFF) [file pone.0069004.s003.tif]

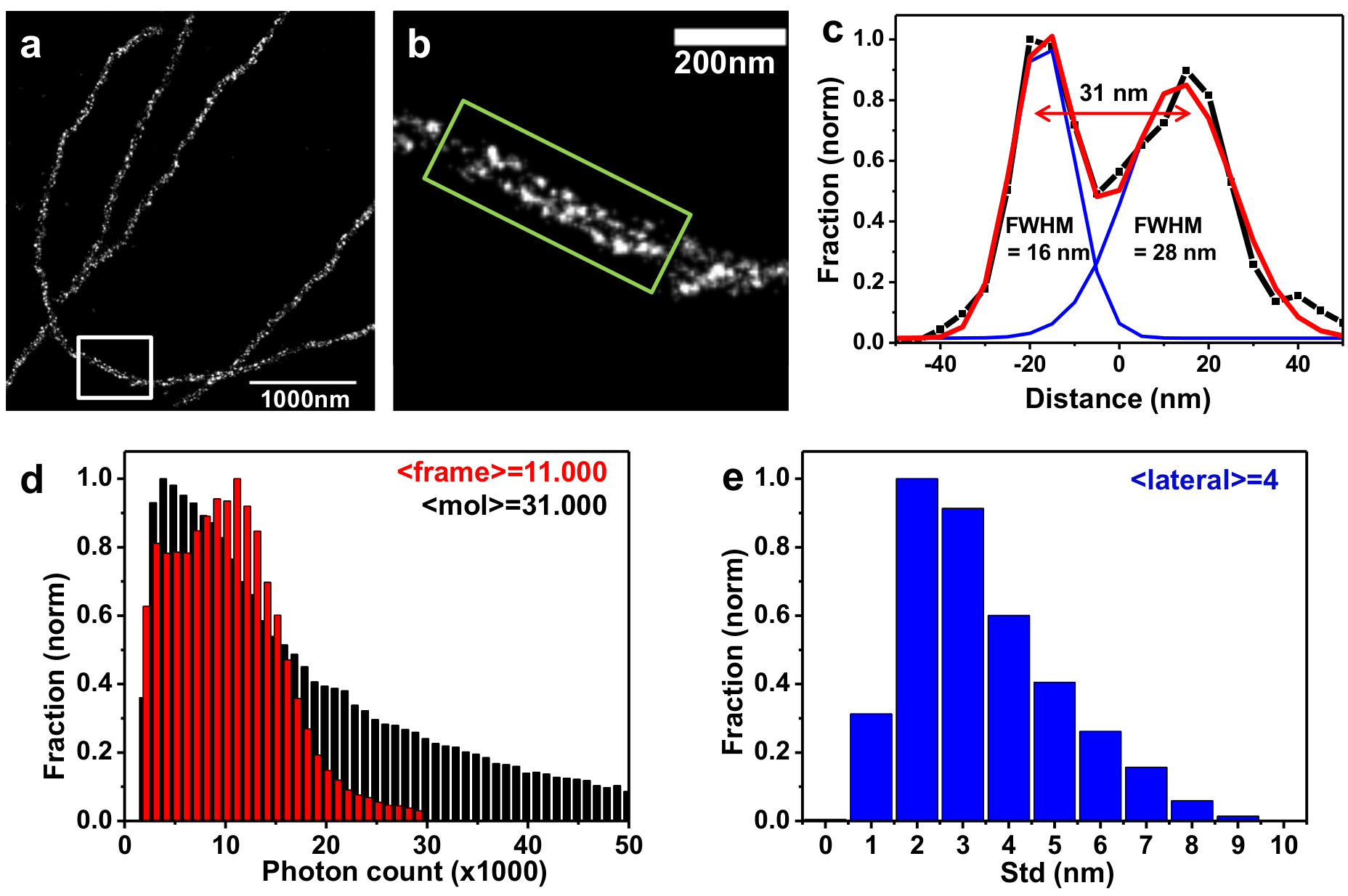

Supplement: Figure S4 — Imaging properties using Pyranose Oxidase. (a) 2D STORM image of a COS-7 cell stained with alpha-tubulin primary and Alexa-647 F(ab’)2 secondary antibodies in “Pyranose Oxidase [23] Buffer” (Buffer #6 in Table S1), and (b) Zoom on the boxed region defined in (a) showing the hollowness of microtubules. (c) Lateral profile measured and averaged over a 500 nm-long microtubule region (green box in (b)) and indicated FWHM values as well as distance between two peaks, showing distances consistent with Figure 3e . (d) Normalized frame (red) and grouped molecules (black) photon count distribution, as well as average values given in the top right corner. (e) Normalized frame localization precision in the lateral direction estimated from the dataset shown in (a) by calculating the standard deviation of repeated localizations (threshold of 5 localizations). The actual localization precision is expected to be closer to the standard error of the mean, but using only peaks on for more than 5 frames would skew the value. (TIFF) [file pone.0069004.s004.tif]

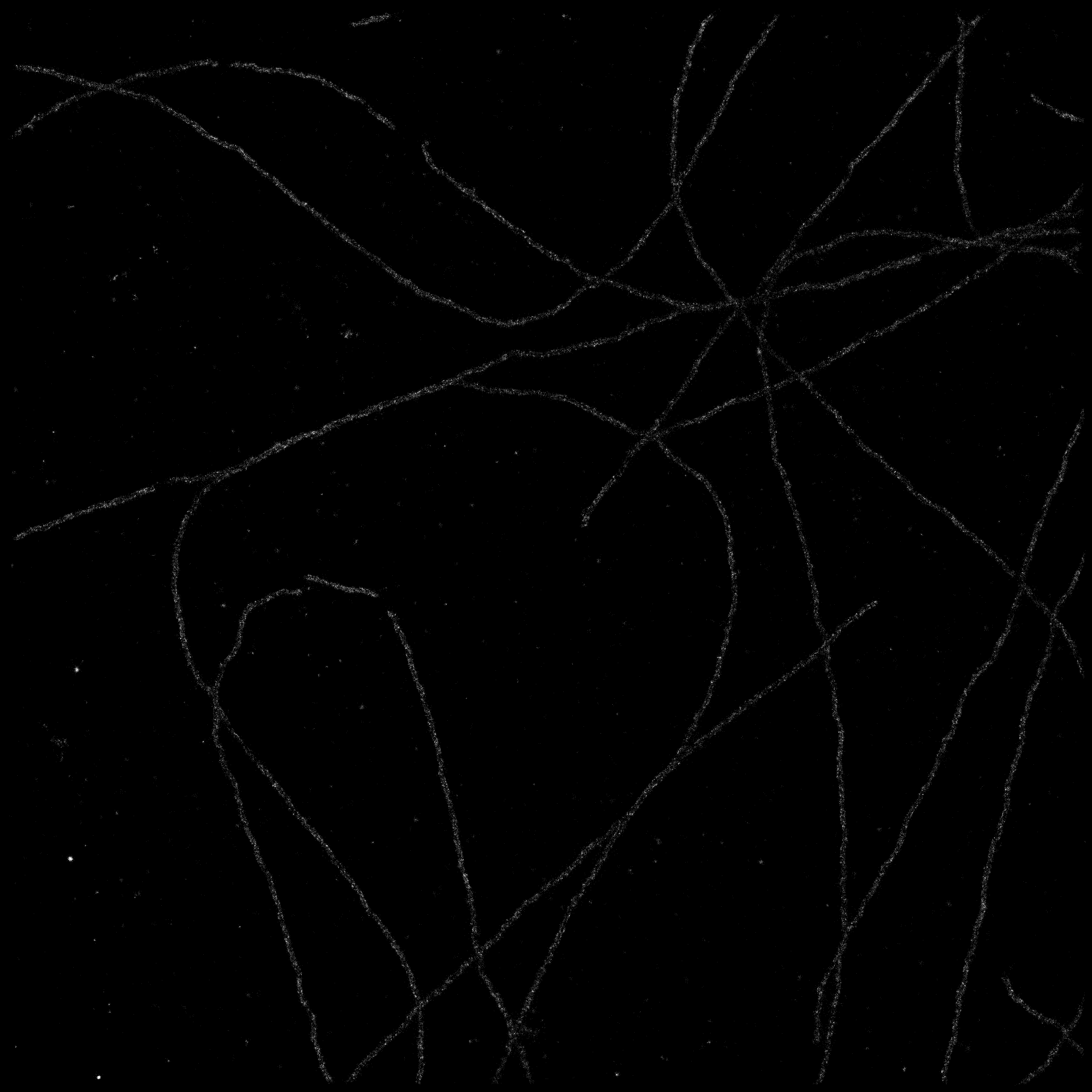

Supplement: Figure S5 — High resolution STORM image of microtubules. STORM image of the data used for Figure 3a–f is displayed by binning the grouped localizations into a regular grid of 3×3 nm. A value of n corresponds to n molecules per pixel. (PNG) [file pone.0069004.s005.png]

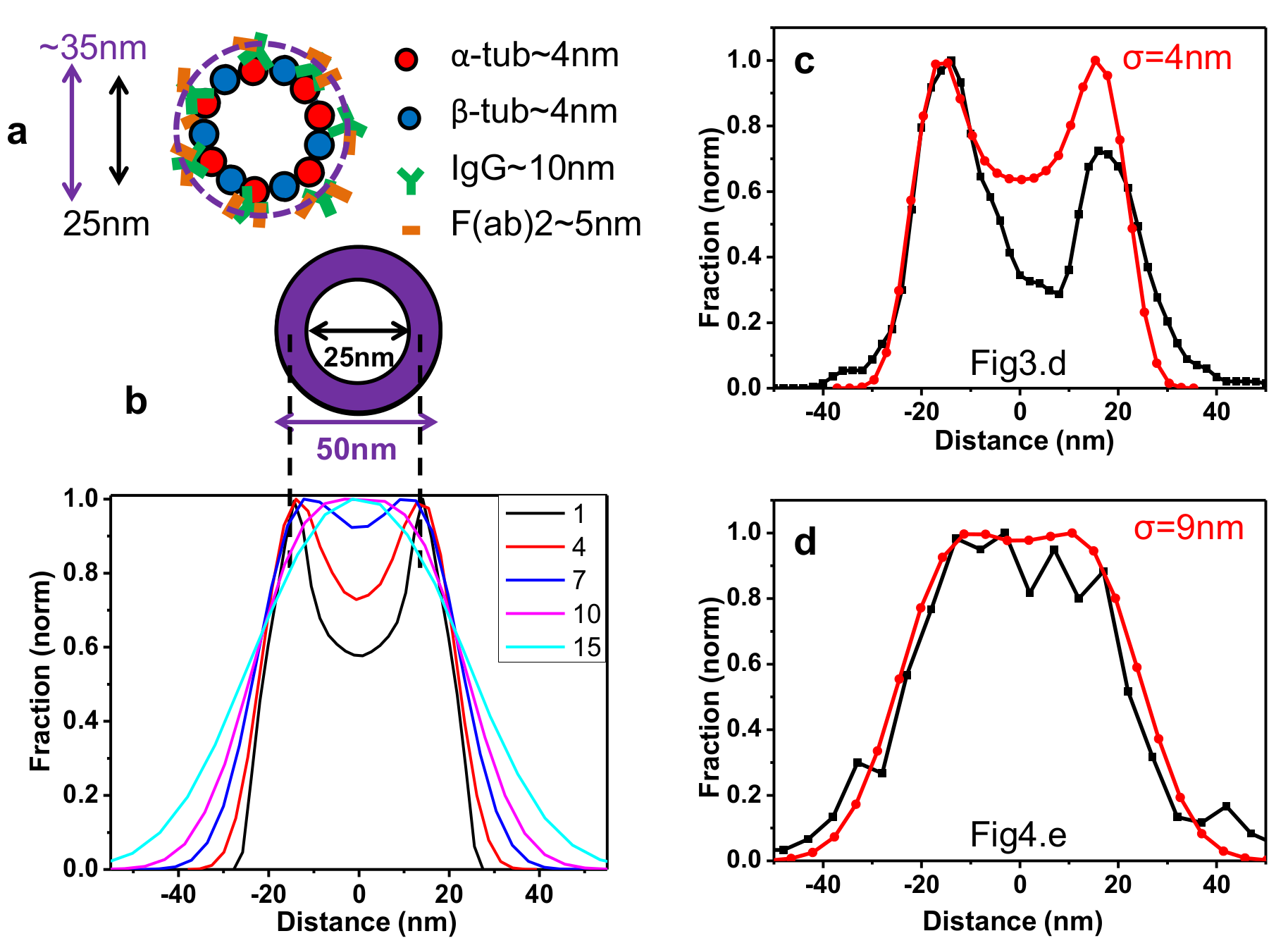

Supplement: Figure S6 — Model of microtubules nanostructure. (a) Schematics of the cross-section of an immuno-labeled microtubule. The epitope of the alpha-tubulin antibody used is located towards the C-terminal end of the protein (manufacturer's datasheet), which was shown by various electron-microscopy studies to be located outside of the tube (reviewed in [25]). We then assume that the primary antibodies are decorated isotropically with smaller secondary antibody fragments, resulting in a hollow labeled tubular structure with respective inner and outer diameter of ∼25 nm, and ∼50 nm. (b) Expected signal from the projection of this 25–50 nm tube assuming different “resolution” (Gaussian blurring of 1–15 nm indicated in sigma). (c) Lateral profile from Figure 3d (black line) compared with the modeled structure assuming a blurring factor of 4 nm (red line) (d) axial profile from Figure 4e (black line) compared with the modeled structure assuming a blurring factor of 9 nm (red line). (TIFF) [file pone.0069004.s006.tiff]

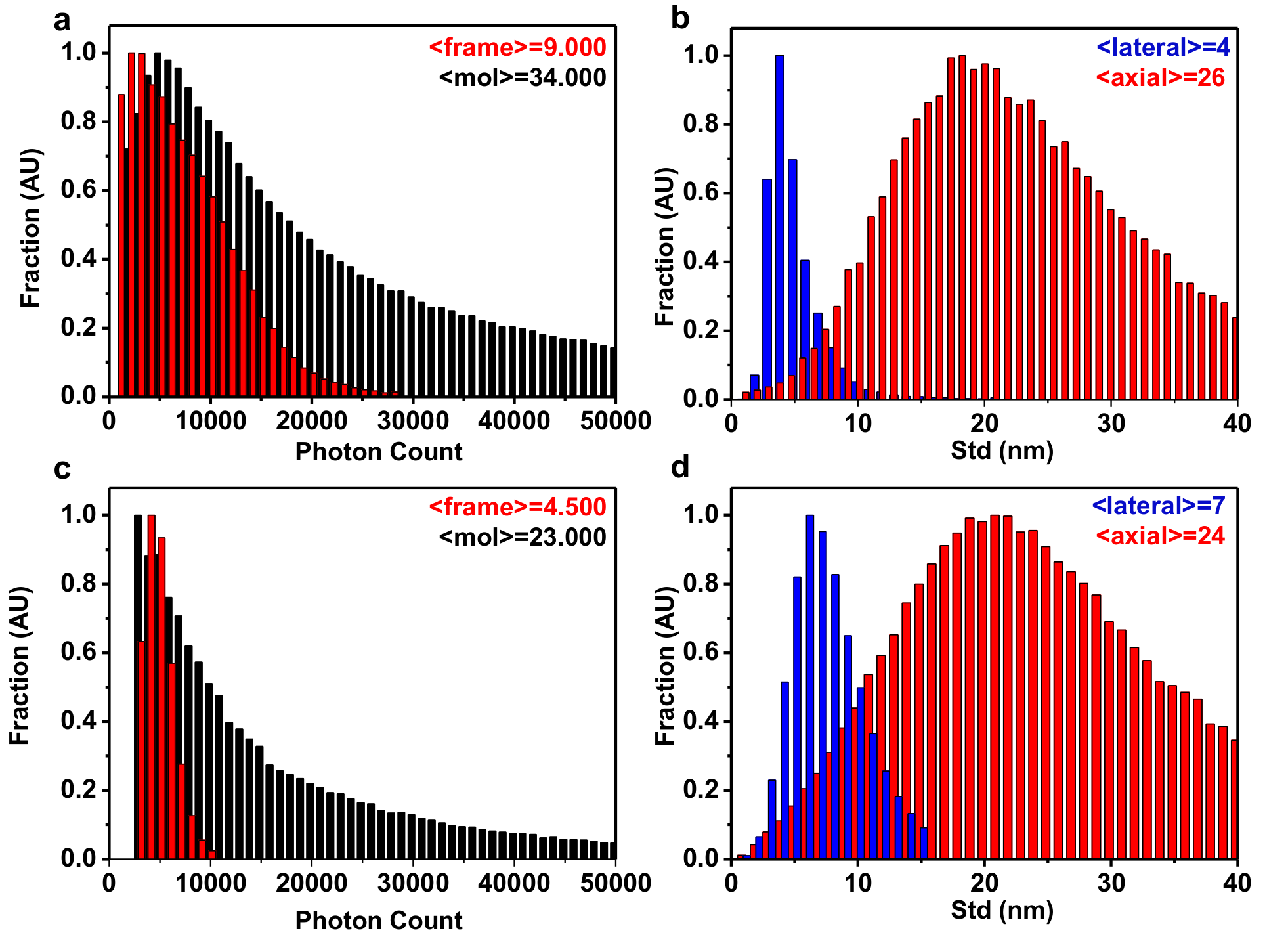

Supplement: Figure S7 — Photon counts & localization precision. (a) Normalized frame and molecule photon counts distribution extracted from the dataset shown in Figure 4a–c (Buffer #4 in Table S1), mean values are indicated in the top right corner. (b) Frame localization precision in both axial and lateral directions estimated from the dataset shown in Figure 4a–c by calculating the standard deviation of repeated localizations (threshold of 5 localizations, see Notes S1 for more details). (c) Frame and molecule photon counts distribution extracted from the dataset shown in Figure 4d–f (Buffer #5 in Table S1), mean values are indicated in the top right corner. (d) Frame localization precision in both axial and lateral directions estimated from the dataset shown in Figure 4d–f by calculating the standard deviation of repeated localizations (threshold of 5 localizations). (TIFF) [file pone.0069004.s007.tiff]

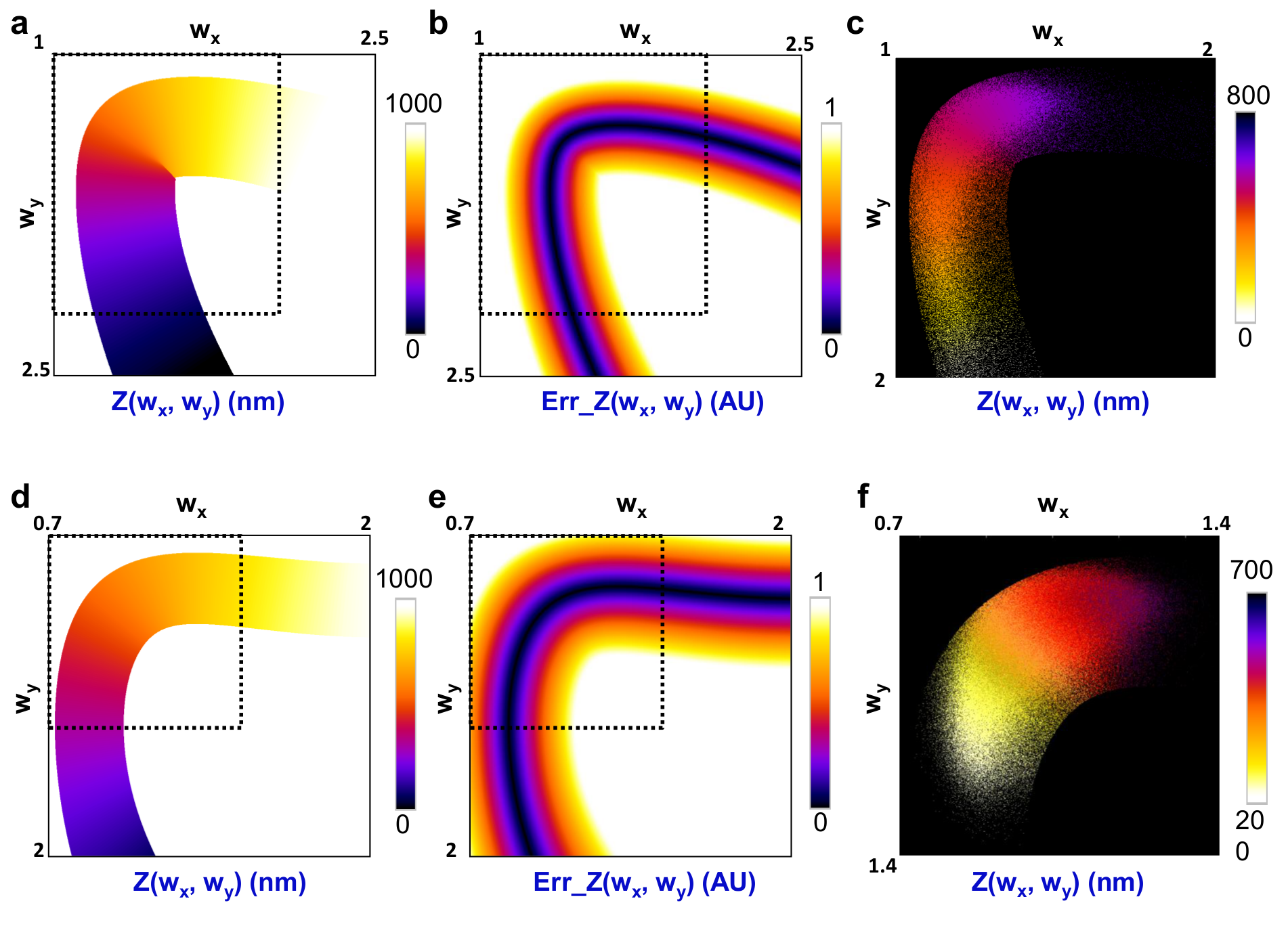

Supplement: Figure S8 — Astigmatism z-calibrations. (a) Estimated z-localization in nm for each couple of (width: wx, height: wy) expressed in pixels between a minimum and a maximum value for the 100× objective obtained from the calibration data (see Data Analysis for more details). (b) Estimated error in the localization associated to the matrix shown in (a). (c) Measured z position vs. measured width and height for the dataset shown in Figure 4a (corresponding region in (a) illustrated by the dotted square). (d) Estimated z localization in nm for each couple of (width: wx, height: wy) expressed in pixels between a minimum and a maximum value for the 60x water objective obtained from the calibration data. (e) Estimated error in the localization associated to the matrix shown in (d). (f) Measured z position vs. measured width and height for the dataset shown in Figure 4e (corresponding region in (d) illustrated by the dotted square). (TIFF) [file pone.0069004.s008.tiff]
