# Supplementary material for: Resolution Doubling in 3D-STORM Imaging through Improved Buffers
Source: PLoS One. 2013 Jul 17;8(7):e69004. doi: 10.1371/journal.pone.0069004 (PMC3714239; doi:10.1371/journal.pone.0069004)
Supplement: Table S1 — Buffers used in this study. (DOCX) [file pone.0069004.s009.docx]

|  | **Used in:** | **Abridged Composition (see Methods for full description)** | **Misc** |
| --- | --- | --- | --- |
| **#1 “BME Buffer”**  **(+/- COT)** | **Figure 1, Figure S1** | **100mM BME + GLOX** |  |
| **#2 “MEA Buffer”**  **(+/- COT)** | **Figure 1** | **100mM MEA + GLOX** |  |
| **#3 “BME+MEA Buffer”**  **(+/- COT)** | **Figure 1, Figure S2** | **10mM MEA+ 50mM BME + GLOX** | **used for testing the influence of Trolox, and Propyl Gallate,** |
| **#4 “BME+MEA+PCA/PCD”**  **(+/- COT)** | **Figure 2, Figure 3,**  **Figure 4d-f, Figure 5**  **Figure S1,** | **10mM MEA+ 50mM BME + PCA/PCD** | **-10pc glucose added in figure 2**  **-used for testing influence of salts**  **-used for testing Cy5 & CF647** |
| **#5 “BME+MEA”**  **in 75% Glycerol”** | **Figure 4a-c** | **10mM MEA+ 50mM BME + PCA/PCD + 2mM COTin 25% PBS - 75% Glycerol** | **Index of refraction = 1.44** |
| **#6 “Pyranose Buffer”** | **Figure S3** | **10mM MEA+ 50mM BME + Pyranose Oxydase +catalase+ 2mM COT in PBS-10% Glucose** |  |

**Table S1: Buffers used in this study**
