# Supplementary material for: Resolution Doubling in 3D-STORM Imaging through Improved Buffers
Source: PLoS One. 2013 Jul 17;8(7):e69004. doi: 10.1371/journal.pone.0069004 (PMC3714239; doi:10.1371/journal.pone.0069004)
Supplement: Notes S1 — Discussion on the factors affecting the variability of STORM measurements. (DOCX) [file pone.0069004.s011.docx]

**Supplementary Notes**

**1)** The main variability during STORM imaging in our hands was due to changes in the pH and oxygen concentration. While pH is easy to measure and to stabilize (see **Main Text**), we tried to minimize the variability due to oxygen concentration by letting the enzymatic systems act on the buffer for about 1h before adding it to the sample to deplete the initial oxygen concentration present in the buffer (See **Imaging buffers preparation**), and then waiting a few more minutes before starting the acquisition (to deplete the oxygen introduced when transferring the buffer). This allowed us to diminish the kinetic aspect of oxygen scavenging, to try to establish a steady-state situation. This could also be the reason why PCA/PCD worked well in our hands, and why we could use a smaller concentration of glucose-oxydase/catalase than generally reported.

In terms of mean photon count variability, the main factor was the threshold on what constitutes a single molecule. We used a fairly high threshold, resulting in detection of molecules that had a minimum photon count of ~ 1000 photons, which somewhat skews the average towards higher values, but was chosen to correspond to the settings used for imaging. Finally, the photon count values we report, even without added COT, are slightly larger than the values generally reported: part of it could be due to the higher threshold used here, but part of it is could also be due to the wavelength (641nm) and filters used. However, we assessed the improvement of performance due to the addition of COT by determining the increase in photon-count between regular and COT-enhanced buffers using the exact same conditions (filters, laser power, camera settings, and data analysis). These ratios are therefore an accurate measure of the improvement that can be obtained by using COT-supplemented buffers on any STORM microscope.

**2)** The concentrations used for the “MEA+BME” buffer were chosen empirically, by balancing the brightness and spontaneous re-activation. We tested a range of different chemicals (MEA, BME, DTT (10-50 mM, Sigma 43816), DTE (10-50 mM – Fisher-Bioreagents BP173), TCEP (1-20 mM – Alfa-Caesar H51864), Glutathione (5-20 mM Sigma-Aldrich G6013) and found that most of them worked well to induce blinking, except Glutathione which did not work at all for Alexa-647. Adding TCEP to another thiol resulted in both reduced ON times and reduced OFF times-which could be useful at low concentration (<10 mM) to increase the speed of imaging without losing too much spatial resolution.

**3)** The quality of a STORM image depends on several factors including the brightness of each molecule (Sample), the collection efficiency of the microscope (Hardware) and the quality of the localization method (Software). We have shown here that COT significantly enhances the brightness of each molecule (see **Figure 1 & 2**). However, such an increase would be useless if it prevented proper cycling of the dyes, and hence decreased the density of localized molecules. We show in **Figure S1** that Alexa-647 displays sustained blinking over long periods of illumination, and that this blinking rate can be controlled with a UV laser. However, the correct conditions for making an appropriate comparison between the buffers with or without COT are difficult to define; thus, we also ensured that there is still a high enough density of molecules to reconstruct our image accurately upon COT addition. To this end, we imaged 2 different areas of the same sample immunostained with alpha-tubulin primary and Alexa-647-labelled secondary antibodies, first with a “BME” buffer without COT (*buffer #1*- see **Table S1**), then with a “BME” buffer containing 2 mM COT, and compared the peak density on straight microtubule segments. Both images were reconstructed from 10,000 raw images with no UV used. As shown in the table below, both buffers gave rise to comparable peak densities that are large enough to reconstruct the continuous tubular structures.

| 100 mM BME - No COT | 1 peak every 1.45nm |
| --- | --- |
| 100 mM BME + 2 mM COT | 1 peak every 1.40nm |

The data shown in **Figure 3** has a higher density of ~ 1.4 molecules / nm for 20,000 frames, even after discarding all lower intensity (< 5,000photons) peaks.

**4)** We define “frame photon counts” as the number of photons measured without doing any grouping, while the “molecule photon count” is the value obtained by adding all the frame photon counts for one molecule after grouping (see grouping procedure in **Data Analysis**).

The localization precision is sometimes estimated from the standard deviation of repeated localizations originating from a single cluster, but that requires having such clusters present in the images, and if too few clusters are present that might skew the result. Moreover, only the average value of the localization precision is usually given. What we show here is a slightly different measure, since it represents the distribution of the standard deviation of the calculated position of all the molecules that appear in more than 5 consecutive frames (~ 5% of the localized molecules in this case). This value should give an accurate measure of the actual localization precision if no grouping is performed. Since we group molecules, the localization precision of our molecules increases, in theory by ***sqrt(n)***, ***n*** being the number of times the molecule is localized. The localization precision can therefore be approximated by the frame localization precision divided by the average number of times a molecule is localized (in the case represented above, around 4-5 times), which would shift the values reported here by approximately a factor of 2.

**5)** When using a non-index-matched buffer, the light emitted by molecules located deep into the sample are subject to two principal aberrations: one is defocus, which means that the object appears further away than it actually is, and the other is spherical aberration, which increases the size of object. Defocus is easy to take into account by adding a linear scaling factor, but spherical aberration is harder to compensate for, though careful calibrations can permit this [27].

Here, we only compensate for defocus by adding a 0.9 scaling factor when using the glycerol+PBS buffer (Buffer #5 of **Table S1**), and spherical aberrations are the main reason behind the broadening as a function of depth observed in **Figure 4**. Using the water immersion objective means that we achieve index matching, rendering a correction scheme unnecessary.

**6)** Influence of ions: since most chemicals required for the buffers need to have their pH adjusted, we measured the influence of some of the ions used (Na^+^ from NaOH, Cl^-^ for HCL, K^+^ from KOH, and I^-^ from KI) and found that the effect of adding up to 50 mM of either Na^+^, K^+^ or Cl^-^ did not affect blinking significantly, but that I^-^ decreased significantly both ON and OFF times, as previously reported [12], making imaging impossible for concentrations over ~ 10 mM.

**7)** We tested whether COT also had an influence on two other far-red dyes: Cy5 (Life Technologies) and CF647 (Biotium) using our “MEA+BME“ buffer (#4 in **Table S1**). In both cases, the mean photon count was significantly improved (~ 3x), and the ability to blink was maintained. We also tested COT on other dyes (Alexa-488, Alexa-546, Alexa-555, all from Life Technologies) in the same buffer conditions, but we could not collect a high enough density of peaks to yield a good STORM image either with or without COT, which prevents us from making a meaningful conclusion on how COT would affect their blinking.

# 8) We introduced astigmatism in the commercial biplane system through de-alignment of the optical element. While this primarily added astigmatism, as can be seen from the shape of the psf, it also added other aberrations. Some of them are actually welcome, since they make the psf more z-dependent, therefore increasing the amount of information that can be extracted. But most are not, especially the high frequency components since they primarily result in blurring the psf in the present signal to noise regime. We therefore expect that a system with a better control of the aberrations would result in even higher localization precisions than the ones achieved here.
